# Oxidative Stress in Chronic Obstructive Pulmonary Disease

**DOI:** 10.3390/antiox11050965

**Published:** 2022-05-13

**Authors:** Peter J. Barnes

**Affiliations:** National Heart and Lung Institute, Imperial College London, London SW5 9LH, UK; p.j.barnes@imperial.ac.uk

**Keywords:** inflammation, cellular senescence, 8-isoprostane, antioxidants, Nrf2

## Abstract

There is a marked increase in oxidative stress in the lungs of patients with COPD, as measured by increased exhaled 8-isoprostane, ethane, and hydrogen peroxide in the breath. The lung may be exposed to exogenous oxidative stress from cigarette smoking and indoor or outdoor air pollution and to endogenous oxidative stress from reactive oxygen species released from activated inflammatory cells, particularly neutrophils and macrophages, in the lungs. Oxidative stress in COPD may be amplified by a reduction in endogenous antioxidants and poor intake of dietary antioxidants. Oxidative stress is a major driving mechanism of COPD through the induction of chronic inflammation, induction of cellular senescence and impaired autophagy, reduced DNA repair, increased autoimmunity, increased mucus secretion, and impaired anti-inflammatory response to corticosteroids. Oxidative stress, therefore, drives the pathology of COPD and may increase disease progression, amplify exacerbations, and increase comorbidities through systemic oxidative stress. This suggests that antioxidants may be effective as disease-modifying treatments. Unfortunately, thiol-based antioxidants, such as N-acetylcysteine, have been poorly effective, as they are inactivated by oxidative stress in the lungs, so there is a search for more effective and safer antioxidants. New antioxidants in development include mitochondria-targeted antioxidants, NOX inhibitors, and activators of the transcription factor Nrf2, which regulates several antioxidant genes.

## 1. Introduction

Chronic obstructive pulmonary disease (COPD) affects about 10% of people of the world’s population over 40 years of age and is increasing, particularly in low- and middle-income countries, as populations age [1]. COPD is now the third-ranked cause of death after cardiovascular disease and stroke, the fifth-ranked cause of chronic disability, and a leading cause of emergency hospital admission [1]. The economic burden of COPD is very high, driven by the costs of hospitalisation for acute exacerbations and the cost of long-term drug therapy [2]. In the UK, COPD costs around GBP 2 billion/year [3]. Costs are expected to rise globally, particularly when the costs of comorbidities are taken into account; COPD is predicted to cost over USD 5 trillion annually by 2030 [4]. Costs rise with increasing disease severity, so a major aim is to prevent disease progression. However, current optimal therapy with inhaled long-acting bronchodilators is symptomatic and does not modify the underlying disease to reduce disease progression [5].

### 1.1. Risk Factors

Cigarette smoking is the main risk factor in high-income countries, and although cigarette consumption is decreasing in these countries, it is rising in low- and middle-income countries (LMIC), especially among women. Over half of the COPD cases in the world occur in non-smokers [6], and exposure to biomass smoke is a risk factor for COPD, especially in women; it has similar characteristics to smoking-related COPD [7], with similar genetic signals [8]. There is increasing epidemiological evidence that ambient outdoor air pollution is an important risk factor with exposure to particulates, nitrogen oxides (NO_x_), and ozone, particularly in large cities in Asia [9]. Household air pollution is also important, especially in LMICs, and accounts for the high prevalence of COPD in women in India [10,11]. Occupational exposure to dust and chemicals may also be an important risk factor affecting particular occupations such as farmers and firefighters [12]. All of these environmental risks impose oxidative stress on the lungs that may overcome antioxidant defences. Diet may also be a risk factor for COPD if there is a deficiency in antioxidants, such as antioxidant vitamins and flavones [13]. Genetic causes of COPD have been more difficult to define, apart from alpha_1_-antitrypsin deficiency, but multiple gene polymorphisms may determine the expression of various endogenous antioxidant genes that may amplify the lung response to exogenous oxidative stress [14].

### 1.2. Inflammation in COPD

COPD is associated with chronic inflammation of the lung, which particularly affects peripheral airways and the lung parenchyma and leads to small airway fibrosis and emphysema, which are progressive [15]. Although inhaled irritants, such as cigarette smoke and particulates, may initiate inflammation, it becomes self-perpetuating and persists even in ex-smokers. Many inflammatory cells are involved in the pathogenesis of COPD, including macrophages (recruited from blood monocytes), neutrophils, eosinophils, and lymphocytes that are recruited into the lungs [16]. Structural cells, including airway epithelial cells, fibroblasts, and endothelial cells also contribute to inflammation [17]. These cells produce multiple mediators, including cytokines that perpetuate and amplify the inflammation in the lungs, and are also important sources of reactive oxygen species (ROS), thus contributing to oxidative stress in the lungs.

There is currently convincing evidence that COPD may be due to accelerated lung ageing with the accumulation of senescent cells [18]. Senescent cells persist in lung tissue and release multiple inflammatory mediators, known as the senescence-associated secretory profile (SASP), which spreads senescence and is very similar to the pattern of mediators that are increased in COPD [19]. Senescent cells also release ROS and, therefore, may be important contributors to oxidative stress in COPD.

It is evident that oxidative stress is a major driving mechanism in COPD and may account for many of the pathophysiological changes that occur [20]. This suggests that antioxidants may be a promising approach to treatment and, by targeting a key driving mechanism, may reduce disease progression and mortality. This review provides an overview of the measurement and generation of oxidative stress in COPD and considers how it may contribute to disease mechanisms, as well as how new antioxidants are being developed as novel therapies for COPD.

## 2. Increased Oxidative Stress in COPD

**Figure 1:**
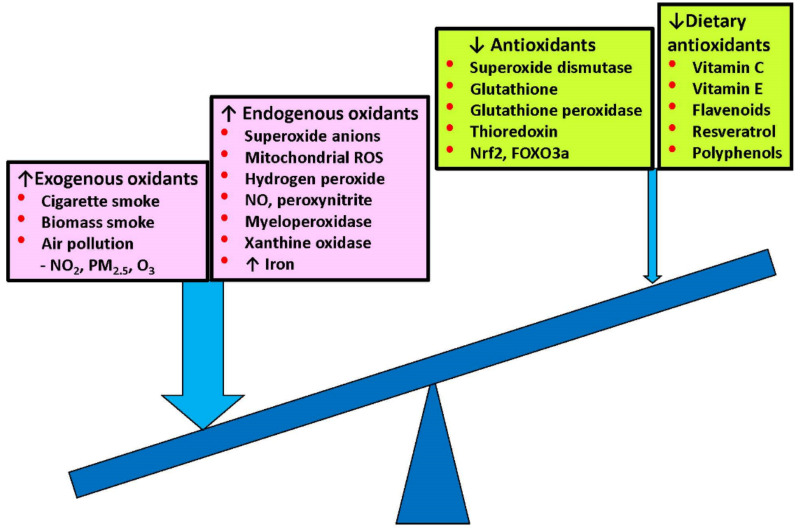
Increased exogenous and endogenous oxidative stress in COPD, which is enhanced by reduced endogenous and dietary antioxidants. ↑: increased; ↓: decreased.

Oxidative stress is increased in COPD patients, even in ex-smokers and never smokers, and is further increased during acute exacerbations. There are several sources of oxidative stress, which is further enhanced by reduced antioxidants (Figure 1).

### 2.1. Exogenous Oxidative Stress

Cigarette smoke exposure is the greatest risk factor for COPD in high-income countries, but only about 20% of smokers develop airway obstruction, indicating that there are likely to be susceptibility factors that may be genetic or epigenetic, which amplify the normal inflammatory response to an inhaled irritant. Exposure to environmental tobacco smoke is also a risk factor for COPD [21]. Outdoor air pollution involves exposure to NOx, particularly NO_2_, small particulate matter (PM_10_, PM_2.5_) from diesel exhaust fumes, and ozone, all of which increase oxidative stress in the lung [9]. Household air pollution is an important source of oxidant exposure, particularly in LMICs, with exposure to biomass smoke from wood and animal dung, which produce high concentrations of small particulates and NO_2_ in poorly ventilated homes [11]. The phenotype of COPD in non-smokers in LMICs is very similar to that seen in smoking COPD and has a similar pattern of inflammation [7]. This indicates that exposure to inhaled irritants is a common factor in producing mucosal inflammation in a susceptible population and that this is likely to be driven by oxidative stress. 

### 2.2. Endogenous Oxidative Stress

Lung inflammation and oxidative stress persist in ex-smokers, indicating that oxidative stress is also generated endogenously. This is likely to be due to the production of ROS by various inflammatory cells within the lungs of COPD patients. Alveolar macrophage numbers are increased over 20-fold in the lungs of COPD patients and are derived from circulating monocytes that are recruited into the lungs by monocyte chemotactic factors produced by structural cells in the lungs. Macrophages from COPD patients are activated and release multiple inflammatory mediators, including ROS as superoxide anions and hydrogen peroxide (H_2_O_2_) [22]. Activated neutrophils are also recruited into the lungs of COPD patients. Peripheral blood neutrophils from COPD patients are also activated and release ROS, particularly during acute exacerbations [23]. Lipid peroxidation is a marker of oxidate stress, and the product 4-hydroxy-2-nonenal (4HNE) is increased in the lung tissue of COPD patients, indicating the local production of ROS [24]. Furthermore, 4HNE is also increased in the plasma of COPD patients during acute exacerbations [25].

### 2.3. Generation of Endogenous ROS

The lungs are exposed to exogenous oxidants in inspired air but also to endogenous ROS generated by mitochondrial respiration and inflammatory responses to inhaled pathogens, such as bacteria and viruses. Mitochondria are dysfunctional in COPD, with leaky membranes, and are increased due to d defect in clearance by mitophagy, resulting in the generation of mitochondrial ROS (mROS), which are major sources of oxidative stress in COPD [26,27]. Inflammatory cells, particularly neutrophils and macrophages that are recruited into the lungs, as well as structural cells, such as airway epithelial cells and fibroblasts, generate endogenous oxidative stress in the lungs. Airway epithelial cells from COPD patients produce intracellular ROS mainly from leaky abnormal mitochondria [28], but also from membrane-bound reduced nicotinamide adenine dinucleotide phosphate (NADPH) oxidases (NOX), from the xanthine/xanthine oxidase system and also from neutrophil-derived myeloperoxidase (MPO) [20]. Some patients with COPD have increased eosinophils, and eosinophil peroxidase is increased in the sputum of COPD patients [29].

Superoxide anions are generated mainly by NOX, and although relatively weak oxidising agents, they are rapidly converted to more damaging ROS species, such as the hydroxyl radical and H_2_O_2_, or the highly reactive peroxynitrite radicals formed when in the presence of nitric oxide (NO) [30]. MPO is released from activated neutrophils and generates very destructive hypochlorous acid, which interacts with tyrosine residues in proteins to form 3-chlorotyrosine, which is increased in the sputum of COPD patients [31]. Intracellular antioxidant defences counteract ROS production to maintain redox balance and, thus, protect cells in the lung. However, in COPD, several endogenous antioxidants are reduced, thus enhancing oxidative stress in the lungs. 

ROS generates reactive carbonyls through lipid peroxidation and glycoxidation of sugars, leading to the formation of aldehydes that cause protein carbonylation [32]. Protein carbonylation, known as ‘carbonyl stress’, is associated with chronic disease and ageing. Protein carbonylation is non-enzymatic and targets specific amino acids, including lysine, arginine, cysteine, and histidine. Protein carbonylation is increased in the lungs of smokers and COPD patients and correlates with disease severity [33]. Protein carbonylation may modify protein function, and this may contribute to disease mechanisms but may also lead to the formation of autoantibodies to the carbonylated proteins. 

### 2.4. Measuring Oxidative Stress in COPD

**Figure 2:**
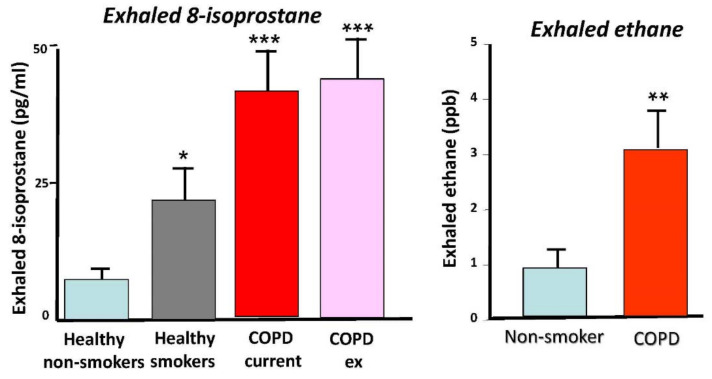
Increased markers of oxidative stress in the breath of COPD patients. Left panel shows increased concentrations of 8-isoprostane in exhaled breath condensate from normal smokers and a greater increase in COPD patients. Ex-smokers have a similar level of 8-isoprostane to active smokers, indicating endogenous oxidative stress from chronic inflammation in the lungs (adapted from ref. [37]). Right panel shows increased exhaled ethane (measured by gas-chromatography mass spectrometry) in COPD patients compared with smokers (adapted from reference [33]). Difference from normal controls: * *p* < 0.05, ** *p* < 0.01, *** *p* < 0.001.

Various approaches have been used to measure oxidative stress in COPD patients. Several biomarkers of oxidative stress have been measured in the breath in order to assess oxidative stress in the lung (Figure 2). Ethane is a volatile product of lipid peroxidation that can be detected by gas chromatography and is increased in exhaled breath of COPD patients, with a positive correlation with disease severity [34]. Exhaled breath condensate has been used to measure various markers of oxidative stress, including H_2_O_2_, malondialdehyde, 4HNE, and 8-isoprostane, showing increases in patients with COPD, compared with smokers without COPD and healthy non-smokers [35,36,37,38]. H_2_O_2_ and 8-isoprostane are further increased during exacerbation [39,40]. Biomarkers of oxidative stress remain increased in ex-smokers, suggesting that they are derived endogenously, most likely from activated inflammatory cells in the lungs [38]. Increased oxidative and nitrative stress generate superoxide anions and NO, respectively, and these rapidly generate highly reactive peroxynitrite, which is detectable in exhaled breath condensate of COPD patients by the specific oxidation of a fluorescent substrate 2′,7′-dichlorofluorescein and is correlated with disease severity [30]. Peroxynitrite nitrates tyrosine residues in proteins and increased nitrotyrosine is increased in induced sputum and lungs of patients with COPD [41,42]. Superoxide anions are also increased in skeletal muscle of patients with COPD and correlate with muscle atrophy and weakness, which are commonly found in severe COPD patients [43].

### 2.5. Reduction in Antioxidants

The increased oxidative stress in COPD may be amplified by a reduction in exogenous antioxidants in the diet and by impaired endogenous antioxidant defences. Glutathione concentrations are reduced in bronchoalveolar lavage fluid from COPD patients with frequent exacerbations, compared with those with stable COPD [44]. Extracellular superoxide dismutase (EC-SOD, SOD3) polymorphisms are seen in COPD, and there is reduced expression around small airways [14,45,46]. Thioredoxin is an important regulator of redox balance and is reduced in COPD [25,47]. 

Nuclear factor erythroid 2-related factor 2 (Nrf2) is a key transcription factor that regulates multiple antioxidant genes and, thereby, protects the lungs against oxidant damage [48]. 

Nrf2 is activated in normal smokers, but its activation by oxidative stress is impaired in COPD as a result of histone acetylation due to histone deacetylase (HDAC)-2 reduction, resulting in reduced antioxidant gene expression [49]. The transcription factor Forkhead box O3a (FOXO3a) also regulates multiple antioxidant genes and is reduced in COPD lungs as a result of phosphoinositide-3-kinase (PI3K) activation [18,50]. 

### 2.6. Iron Overload

Iron is increased in the lungs of COPD patients and iron overload in lung cells generates ROS production, particularly mROS and lipid peroxidation [51]. Iron accumulation may lead to a specific form of non-apoptotic cell death known as ferroptosis. The hepcidin–ferroportin iron transporter mechanism is abnormal in COPD alveolar macrophages, which may lead to the accumulation of iron and the generation of ROS [52].

## 3. Effects of Oxidative Stress in COPD

**Figure 3:**
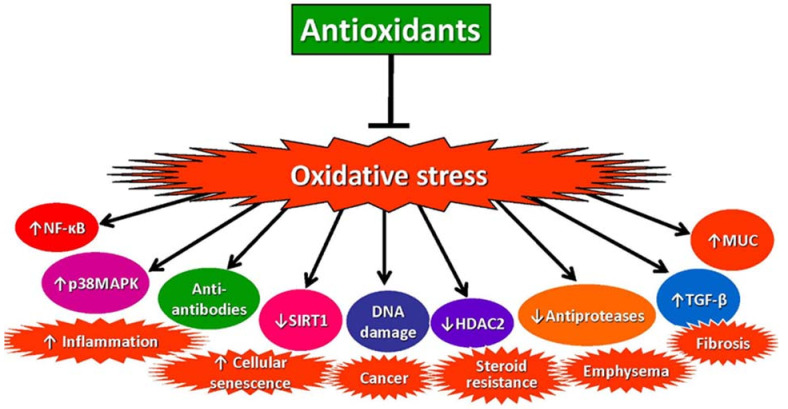
Increased oxidative stress drives the pathology of COPD through several mechanisms. These include the proinflammatory transcription factor nuclear factor-KB (NF-κB), p38 mitogen-activated protein kinase (MAPK), generation of autoantibodies to carbonylated proteins, reduced expression of sirtuin-1 (resulting in cellular senescence), DNA damage (increasing lung cancer risk), reduced histone deacetylase (HDAC)-2 expression (inducing steroid resistance), reduced activity of antiproteases (resulting in emphysema), increased release of transforming growth factor(TGF)-β (resulting in small airway fibrosis), and increased expression of mucin genes (MUC) (causing mucus hypersecretion. ↑: increased; ↓: decreased; ┴: block.

There is compelling evidence that increased oxidative stress is a major driver of the pathophysiology of COPD through several different mechanisms [20,53,54] (Figure 3).

### 3.1. Increased Inflammation

Over 100 different mediators are secreted in COPD, including multiple cytokines and chemokines, which amplify and perpetuate lung inflammation [55]. Oxidative stress activates the intracellular signalling pathways that lead to the synthesis and release of these inflammatory mediators, the proinflammatory transcription factor nuclear factor-κB (NF-κB) and signalling molecules such as Ras/Rac, p38 mitogen-activated protein kinase (MAPK), Jun-*N*-terminal kinase (JNK), PI3 kinase, and protein tyrosine phosphatases. NF-κB expression and activation are increased in COPD, particularly in airway epithelial cells and macrophages, and activated by ROS [56]. Oxidative stress also activates transforming growth factor(TGF)-β signalling, which itself induces oxidative stress [57] and is likely to account for small airway fibrosis, which is believed to be the initial lesion of COPD and accounts for early disease progression [58]. TGF-β has an inhibitory effect on Nrf2, which increases oxidative stress through the suppression of endogenous antioxidants [59]. Oxidative stress increases the expression of matrix metalloproteinase (MMP)9, breaking down elastin fibres, which may be an important mechanism of emphysema, and further enhances elastolysis through inactivation of α1-antitrypsin and increased neutrophil elastase activity [60].

### 3.2. Corticosteroid Resistance

Chronic inflammation in COPD lungs is not significantly suppressed by corticosteroids, in contrast to asthma. The corticosteroid resistance may be due to oxidative stress, which inhibits the expression and activity of HDAC-2, which is required for the suppression of proinflammatory genes that are activated by histone acetylation [61]. Oxidative stress reduces HDAC-2 activity and expression through the activation of PI3K-δ, leading to phosphorylation and ubiquitination of this epigenetic regulatory enzyme [62], peroxynitrite, which is increased in COPD lungs, also inactivates HDAC-2 by tyrosine nitration and ubiquitination [63]. The reduction in HDAC-2 by oxidative stress prevents the acetylation of glucocorticoid receptors, which is necessary for the inhibition of NF-κB that mediates the anti-inflammatory effects of corticosteroids [64].

### 3.3. Accelerated Lung Ageing and Cellular Senescence

There is growing evidence that COPD is associated with accelerated lung ageing and the accumulation of senescent cells in the lungs, including epithelial cells, fibroblasts, and endothelial cells [18]. Oxidative stress accelerates telomere shortening, as telomeric DNA is particularly sensitive to oxidative DNA damage [26], leading to the activation of DNA damage response that results in cell-cycle arrest through the activation of p53, which activates the cyclin kinase inhibitor p21^CIP1^. Oxidative stress also activates the cyclin kinase inhibitor p16^INK4^ stress-induced senescence pathway [26]. Oxidative stress also activates the PI3K–mammalian target of rapamycin (mTOR) signalling, which is activated in COPD lungs [65,66]. This results in increased microRNA-34a, which inhibits the expression of sirtuin-1 and sirtuin-6 mRNA and protein [67,68]. Oxidative stress also increases miR-570, which also inhibits sirtuin-1 expression, but via a p38 MAPK–cJUN–AP1 pathway [69]. The key role of miR-34a and -570 in COPD is demonstrated by the reversal of cellular senescence and cell-cycle arrest in small airway epithelial cells by specific inhibitors (antagomiRs) of these microRNAs. Reduction in sirtuin-1 and sirtuin-6 enzyme activity and expression results in increased acetylation of NF-κB and increased inflammation, indicating that cellular senescence may drive inflammation in COPD. The reduction in sirtuin-1 also contributes to mitochondrial dysfunction, impaired DNA repair, and defective autophagy. In addition, decreased sirtuin-1 and sirtuin-6 impair the function of the transcription factors FOXO3a and Nrf2, respectively, which decreases the expression of multiple antioxidant genes, leading to increased oxidative stress which drives further senescence. Oxidative stress reduces autophagy through activation of PI3K–mTOR signalling and a reduction in sirtuin-1, resulting in the accumulation of damaged proteins and organelles, including mitochondria [70,71].

### 3.4. Autoimmunity

There is increasing evidence indicating that autoimmunity may play a role in amplifying and perpetuating COPD, especially in severe diseases, and may account for the persistence of the disease after smoking cessation [72]. Autoantibodies against epithelial and endothelial cells and against collagen and cytokeratin have been detected in COPD patients and appear to increase with disease severity [73]. Oxidative stress may cause carbonyl stress, which creates neoantigens against which autoantibodies may develop. Autoantibodies against carbonyl-modified proteins have been detected n COPD patients and, since these may be complement-fixing, this may contribute to lung parenchymal damage [33].

### 3.5. DNA Damage

Oxidative stress directly damages DNA, especially the DNA in telomeres [26]. Additionally, 8-hydroxy-2-deoxyguanosine is a biomarker of oxidative damage of DN and is increased in the peripheral lungs of normal smokers and patients with COPD, reflecting the increased oxidative stress in the lungs [74]. Apurinic/apyradymic (AP) sites indicate the repair of oxidised DNA bases, and there are normally efficient DNA repair mechanisms. In normal smokers, there is an increase in AP sites in the lungs, signifying active DNA repair, but this is significantly reduced in COPD patients, showing defective DNA repair in COPD. The nuclear expression of the double-stranded DNA repair protein Ku86 is significantly reduced in COPD lungs, compared with normal smokers, demonstrating that there is a defect in DNA repair in COPD [74]. In mice exposed to cigarette smoke, there is a reduction in Ku86, indicating an impairment in the DNA repair machinery with oxidative stress; this is also seen in human small airway epithelial cells exposed to H_2_O_2_. This defect in DNA repair as a result of oxidative stress is likely to contribute to the marked increased prevalence of lung cancer in patients with COPD, compared with smokers without airway obstruction [75].

### 3.6. Mucus Secretion

Oxidative stress stimulates mucus secretion and the expression of mucin (MUC)5AC, which contributes to airway narrowing in COPD [76]. Importantly, oxidative stress markedly increases mucus cross-linking, making mucus more viscous and difficult to clear [77] and may account for the widespread mucus plugging seen in some patients with severe COPD [78]. 

## 4. Strategies for Reducing Oxidative Stress

As oxidative stress is a major driving mechanism for the pathophysiology of COPD, antioxidant therapies should be of great benefit in COPD [79,80,81]. However, antioxidants are not used routinely in the current management of COPD, indicating that currently available antioxidants drugs are not effective. Several classes of antioxidants have been developed, and there are several new drugs in preclinical and clinical development (Table 1).

### 4.1. Dietary Antioxidants

Although epidemiological studies have linked diets low in antioxidants with poor lung function, and they are recognised as risk factors for the development of COPD, antioxidant vitamins have not been found to benefit established COPD [13]. Dietary antioxidants include vitamin C (ascorbic acid), vitamin E (α-tocopherol), resveratrol (found in red-skinned fruits and red wine), and flavonoids, such as quercetin (found in many fruits and vegetables), but dietary antioxidants do not improve lung function or clinical features of COPD [82,83]. There is retrospective evidence that a Mediterranean diet, rich in dietary antioxidants, may protect against the development of COPD, but many confounding factors make this difficult to interpret [84]. Resveratrol has been shown to reduce ROS and inflammatory mediator release from airway epithelial cells from COPD patients in vitro, and in rats in vivo, it reduces neutrophilic inflammation induced by lipopolysaccharide [85,86]. However, resveratrol has poor oral bioavailability, which has led to the development of more potent and orally bioavailable analogues, known as sirtuin-activating compounds. Inhaled resveratrol reduces accelerated lung ageing in a telomerase-deficient mouse model [87]. (−)-Epigallocatechin is a polyphenol found in green tea that activates FOXO3a, a transcription factor that regulates antioxidant genes, such as SOD and catalase [88]. (−)-Epigallocatechin reduces oxidative stress and neutrophil inflammation in rats exposed to cigarette smoke in vivo and in human airway epithelial cells in vitro [89].

### 4.2. Thiol-Based Antioxidants

*N*-Acetylcysteine (NAC was developed as a mucolytic agent and is a thiol compound that, as it breaks down mucin disulphide, cross-links to reduce mucus viscosity. It was found to have antioxidant effects by increasing glutathione concentrations, which are reduced in COPD [90]. Several small clinical studies in COPD patients showed a reduction in exacerbations [91], although a large clinical trial (n = 523) of low-dose (600 mg orally) NAC (Bronchitis Randomised on NAC Study (BRONCUS)) failed to show any reduction in exacerbation or to reduce disease progression; nevertheless, there was a significant effect in patients who were not treated with inhaled corticosteroids (ICS) [92]. A larger study (>1000) in Chinese COPD patients with a higher dose of NAC (600 mg twice daily) showed a small reduction (~20%) in exacerbation rate [93], which was greatest in current smokers and in those not treated with ICS [94].

Carbocisteine, a similar thiol mucolytic drug that has antioxidant effects, also reduces the exacerbations in COPD patients not treated with ICS [95], which was confirmed in a meta-analysis of four placebo-controlled studies [96]. Erdosteine, another thiol antioxidant, reduces mild (but not moderate or severe) exacerbations [97] and was confirmed in a meta-analysis [98]. Thiol-based antioxidants have only a modest effect in reducing exacerbations and have no effects on lung function or quality of life [99,100]. It is not certain whether any clinical benefits result from their mucolytic actions, by increasing mucociliary clearance, or due to their antioxidant effects on the lungs. As oxidative stress is high in the lungs, systemic administration may not be able to counteract the high local oxidative stress, so they may be better delivered directly into the lungs. NAC has also been given by nebulisation and a lysine derivative nacystelyn by dry powder inhaler, but they may induce bronchoconstriction, and there is no evidence of any clinical benefit in COPD. A major problem with thiol-based antioxidants is their rapid inactivation by the high level of oxidative stress in COPD lungs, prompting a search for more stable antioxidants.

### 4.3. Antioxidant Mimetics

Antioxidant mimetics restore depleted endogenous antioxidants, such as SOD, catalase, and glutathione peroxidase (GPx) [101]. Overexpression of Cu-Zn SOD in mice protects against the development of emphysema after cigarette smoke exposure, suggesting that enhancing SOD activity may be beneficial in COPD [102]. SOD mimetics include metalloporphyrins, such as AEOL 10113 and AEOL 10150, and manganese-containing molecules, such as M40419. These drugs are effective in different animal in vivo models of oxidative stress and reduce the inflammatory response to cigarette smoke in mice [103]. GPx mimics catalyse the breakdown of H_2_O_2_ and include selenium and non-selenium containing antioxidant enzymes_._ GPx transgenic mice are protected against the development of inflammation and emphysema after cigarette smoke exposure, whereas GPx gene knockout increases the inflammatory response to smoke, suggesting that GPx mimetics may be useful in COPD [104]. Ebselen, a GPx mimetic, reduces the pulmonary inflammatory response and endothelial dysfunction in cigarette smoke-exposed mice [105], but no clinical studies on COPD have been reported.

### 4.4. NOX Inhibitors

Nicotinamide adenine dinucleotide phosphate (NADPH) oxidase (NOX) is a membrane-bound molecular complex that is likely to be a major source of ROS in COPD via the generation of superoxide anions. NOX exists in several isoforms, including NOX1-5 and the dual oxidases Duox1 and 2 [106]. Several NOX inhibitors have been identified, although it has proved difficult to discover selective inhibitors [107]. Apocynin, a non-selective NOX inhibitor, reduces the inflammatory response to cigarette smoke in mice in vivo [108]. Acute administration of nebulised apocynin reduces exhaled H_2_O_2_ concentrations in exhaled breath condensate in COPD patients [109], but no longer-term studies have been reported. Setanaxib (GKT137831) is a dual NOX1/4 inhibitor now in clinical trials but has not yet been studied in COPD.

### 4.5. Peroxidase Inhibitors

Myeloperoxidase (MPO) is markedly increased in COPD sputum, reflecting neutrophil degranulation in the lungs [110]. AZD5904, a potent irreversible MPO inhibitor, reduces oxidative stress and the development of emphysema in cigarette smoke-exposed guinea pigs [111]. Although in clinical studies the drug is well tolerated in human volunteers, it was discontinued for unknown reasons.

### 4.6. Inhibiting Nitrative Stress

As discussed above, superoxide anions combine rapidly with NO to form highly reactive peroxynitrite ions, which form 3-nitrotyrosine adducts in proteins, which may lead to functional changes. NO may be produced by type 1 NOS (also known as neuronal NOS), which is induced by oxidative stress in alveolar epithelial cells of COPD patients [112]. Mice exposed to cigarette smoke in vivo express inducible NOS (iNOS, type 2 NOS) and iNOS gene deficiency, as well as selective iNOS inhibitors, protect against the development of emphysema [113]. Aminoguanidine is a relatively selective inhibitor of iNOS. When given via nebuliser to COPD patients, there is a significant reduction in both central and peripheral exhaled NO, although it does not completely block exhaled NO, suggesting that neuronal NOS may be responsible and indicating that selective iNOS inhibitors may not completely reduce peroxynitrite in COPD [114].

### 4.7. Mitochondria-Targeted Antioxidants

Mitochondria are dysfunctional in COPD, with an increase in mitochondrial mass, fusion, and increased membrane leakiness due to impairment of mitophagy, which normally removes damaged mitochondria and may itself be impaired by oxidative stress [26,115]. These dysfunctional mitochondria are major sources of ROS (mROS) in COPD [116,117]. Mitochondria-targeted (mt) antioxidants have been designed to selectively target mitochondria and are based on the structure of ubiquinone; thus, they are concentrated 50–100-fold in mitochondria. These mt-antioxidants appear to be more effective than conventional antioxidants in several animal models of ageing [118]. Several mt-antioxidants, including mitoQ, mito-TEMPO, pyrroloquinoline quinone, and SkQ1, are in clinical trials for several age-related diseases. Cigarette smoke extract induces mitochondrial dysfunction and release of mROS in human airway epithelial cells in vitro, and this is inhibited by mito-TEMPO [119]. In mice exposed to ozone over 6 weeks, oral mitoQ treatment reduces airway hyperresponsiveness, neutrophilic inflammation, and lung inflammatory mediators [116]. Although mt-antioxidants such as mitoQ are available in health food shops, no clinical studies in COPD patients have so far been reported.

### 4.8. Nrf2 Activators

As discussed above, Nrf2 regulates multiple antioxidant genes and translocates to the nucleus after dissociation from Kelch-like ECH-associated protein 1 (Keap1) to bind to antioxidant response elements in several antioxidant genes. However, in cells from COPD patients, Nrf2 fails to activate antioxidant genes in response to ROS, as in normal cells, which may be due to its acetylation as a result of reduced HDAC-2 activity [49]. Heme oxygenase-1 (HO-1) is one of the antioxidant genes regulated by Nrf2 and generates carbon monoxide and biliverdin, which is converted to the antioxidant bilirubin [120]. Delivery of recombinant HO-1 in mice attenuates the development of emphysema induced by elastase, suggesting that Nrf2 activators might be effective therapy [121]. Treatment of fibroblasts from COPD patients with hemin to activate HO-1 results in reduced cellular senescence, improved mitochondrial function, and reduced ROS production [122] Since Nrf2 activates many other antioxidant genes in addition to HO-1, this suggests that activators of Nrf2 and inhibitors of Keap1 might be even more effective against oxidative stress in COPD. Several electrophilic modifiers of Keap1 have been identified, and some have been tested in clinical studies. Sulforaphane is an isothiocyanate compound found in raw cruciferous vegetables, such as broccoli and Brussel sprouts, and forms thiacyl adducts with Cys residues of Keap1, to release Nrf2 to translocate to the nucleus, resulting in the activation of antioxidant genes. However, sulforaphane has poor specificity and also has toxic effects on cells. A four-week clinical trial of sulforaphane in COPD patients failed to increase antioxidant gene targets of Nrf2 (such as HO-1) or to reduce oxidative stress and inflammation [123]. Bardoxolone methyl is a synthetic triterpenoid, which is more potent than sulforaphane and is effective in a cigarette smoke-exposed mouse model of COPD [124]. However, a Phase 3 clinical trial in renal disease was terminated due to cardiovascular side effects and increased mortality [125]. Dimethyl fumarate (BG-12) is an Nrf2 activator that has been approved for use in multiple sclerosis, although side effects such as flushing, nausea, and diarrhoea are reported [126]. An inhaled microparticulate formulation of BG-12 has recently been developed, but no results in animal models of COPD have been reported [127]. These Nrf2 activators all lack specificity and have several adverse effects, so there is a search for more specific activators such as. drugs that interfere with protein–protein interactions between Nrf2 and Keap1. BTB and CNC homology 1 (Bach1) is a transcription factor that inhibits Nrf2 and is increased in COPD. Inhibition of Bach1 is a potential novel strategy for increasing Nrf2 and antioxidants in COPD [128].

**Table 1:** Antioxidants for COPD.

Antioxidant Type	Examples	Studies in COPD
Thiol antioxidants	*N*-Acetylcysteine Carbocisteine Erdosteine Inhaled glutathione	Reduced exacerbations [91,92,93] Reduced exacerbations [95,96] Reduced exacerbations [97,98] Not tested
Dietary antioxidants	Vitamin C (ascorbic acid) Vitamin E (α-tocopherol) Resveratrol (−)-Epigallocatechol	No controlled clinical trials Anti-inflammatory in vitro [85] Not tested
SOD Mimetics	AEOL 10150	Effective in animal models [103]
GPx Mimetics	Ebselen	Effective in animal models [104]
NOX inhibitors	Apocynin Setanaxib (GKT137831)	Reduces inflammation [108] No studies
Myeloperoxidate inhibitors	AZD 5904	Effective in animal models [111]
iNOS inhibitors	L-NIL	Effective in animal studies [113]
Mitochondria-targeted antioxidants	mitoQ, mitoTEMPO SkQ1	Effective in vitro [116,119] No clinical studies
Nrf2 activators	Sulforaphane Bardoxolone methyl Dimethylfumarate (BG-12)	Clinical trial negative [124] Effective in animal models Not tested

Abbreviations: SOD: superoxide dismutase; GPx: glutathione peroxidase; NOX: NADPH oxidase; iNOS: inducible nitric oxide synthase; L-NIL: L-N^6^-(1-iminoethyl)lysin; Nrf2: nuclear erythroid-2 related factor 2.

## 5. Conclusions

Exogenous oxidative stress from cigarette and biomass smoke exposure and air pollution, endogenous oxidative stress from activated inflammatory cells in the lungs, and reduced antioxidants all lead to a high level of oxidative stress in the lungs. This is a major factor driving the pathophysiology of COPD and its progression, as well as amplifying acute exacerbations. Increased oxidative stress in the lungs of COPD patients results in chronic inflammation, reduced anti-inflammatory effects of corticosteroids, cellular senescence and accelerated lung ageing, autoimmunity, fibrosis of peripheral airways, and mucus hypersecretion. Systemic oxidative stress may contribute to many of the comorbidities associated with COPD.

Antioxidant therapy is, therefore, a logical approach to the treatment of COPD and should be effective in preventing disease progression and exacerbations. Although several different approaches to reducing oxidative stress in COPD have been investigated in animal models and COPD cells in vitro, clinical studies have been limited. Thiol based antioxidants, such as *N*-acetylcysteine, have had disappointing clinical effects, as they are probably inactivated in the lungs, so there is a need to study more effective antioxidants, such as mt-antioxidants, as mitochondria are major sources of ROS in COPD and activators of Nrf2, which should restore impaired endogenous antioxidants. The development of more effective strategies to reduce oxidative stress in COPD patients is an important priority for the future.

## References

[1] GBD Chronic Respiratory Disease Collaborators (2020). Prevalence and attributable health burden of chronic respiratory diseases, 1990–2017: A systematic analysis for the Global Burden of Disease Study 2017. Lancet Resp. Med..

[2] Iheanacho I., Zhang S., King D., Rizzo M., Ismaila A.S. (2020). Economic burden of chronic obstructive pulmonary disease (COPD): A systematic literature review. Int. J. COPD.

[3] British Lung Foundation (BLF) COPD 2022. https://statistics.blf.org.uk/copd.

[4] Bloom D.E., Cafiero E.T., Jané-Llopis E., Abrahams-Gessel S., Bloom L.R., Fathima S., Feigl A.B., Gaziano T., Mowafi M., Pandya A. (2011). The Global Economic Burden of Non-Communicable Diseases.

[5] Singh D., Agusti A., Anzueto A., Barnes P.J., Bourbeau J., Celli B.R., Criner G.J., Frith P., Halpin D.M.G., Han M. (2019). Global strategy for the diagnosis, management, and prevention of chronic obstructive lung disease: The GOLD science committee report 2019. Eur. Respir. J..

[6] Salvi S.S., Barnes P.J. (2009). Chronic obstructive pulmonary disease in non-smokers. Lancet.

[7] Salvi S.S., Brashier B.B., Londhe J., Pyasi K., Vincent V., Kajale S.S., Tambe S., Mandani K., Nair A., Mak S.M. (2020). Phenotypic comparison between smoking and non-smoking chronic obstructive pulmonary disease. Respir. Res..

[8] Shrine N., Guyatt A.L., Erzurumluoglu A.M., Jackson V.E., Brian D.H., Melbourne C.A., Batini C., Fawcett K.A., Song K. (2019). New genetic signals for lung function highlight pathways and chronic obstructive pulmonary disease associations across multiple ancestries. Nat. Genet..

[9] Adamkiewicz G., Liddie J., Gaffin J.M. (2020). The respiratory risks of ambient/outdoor air pollution. Clin. Chest Med..

[10] Sood A., Assad N.A., Barnes P.J., Churg A., Gordon S.B., Harrod K.S., Irshad H., Kurmi O.P., Martin W.J., Meek P. (2018). ERS/ATS workshop report on respiratory health effects of household air pollution. Eur. Respir. J..

[11] Balmes J.R. (2019). Household air pollution from domestic combustion of solid fuels and health. J. Allergy Clin. Immunol..

[12] De Matteis S. (2022). Occupational causes of chronic obstructive pulmonary disease: An update. Curr. Opin Allergy Clin. Immunol..

[13] Scoditti E., Massaro M., Garbarino S., Toraldo D.M. (2019). Role of diet in chronic obstructive pulmonary disease prevention and treatment. Nutrients.

[14] Du Y., Zhang H., Xu Y., Ding Y., Chen X., Mei Z., Ding H., Jie Z. (2019). Association among genetic polymorphisms of GSTP1, HO-1, and SOD-3 and chronic obstructive pulmonary disease susceptibility. Int. J. COPD.

[15] Barnes P.J., Burney P.G.J., Silverman E.K., Celli B.R., Vestbo J., Wedzicha J.A., Wouters E.F.M. (2015). Chronic obstructive pulmonary disease. Nat. Rev. Primers.

[16] Agustí A., Hogg J.C. (2019). Update on the pathogenesis of chronic obstructive pulmonary disease. N. Engl. J. Med..

[17] Barnes P.J. (2017). Cellular and molecular mechanisms of asthma and COPD. Clin. Sci..

[18] Barnes P.J., Baker J., Donnelly L.E. (2019). Cellular senescence as a mechanism and target in chronic lung diseases. Am. J. Respir. Crit. Care Med..

[19] Kumar M., Seeger W., Voswinckel R. (2014). Senescence-associated secretory phenotype and its possible role in chronic obstructive pulmonary disease. Am. J. Respir. Cell Mol. Biol..

[20] Kirkham P.A., Barnes P.J. (2013). Oxidative stress in COPD. Chest.

[21] Yin P., Jiang C.Q., Cheng K.K., Lam T.H., Lam K.H., Miller M.R., Zhang W.S., Thomas G.N., Adab P. (2007). Passive smoking exposure and risk of COPD among adults in China: The Guangzhou Biobank Cohort Study. Lancet.

[22] Schaberg T., Klein U., Rau M., Eller J., Lode H. (1995). Subpopulations of alveolar macrophages in smokers and nonsmokers: Relation to the expression of CD11/CD18 molecules and superoxide anion production. Am. J. Respir. Crit. Care Med..

[23] Noguera A., Batle S., Miralles C., Iglesias J., Busquets X., Macnee W., Agusti A.G. (2001). Enhanced neutrophil response in chronic obstructive pulmonary disease. Thorax.

[24] Rahman I., Van Schadewijk A.A., Crowther A.J., Hiemstra P.S., Stolk J., Macnee W., De Boer W.I. (2002). 4-Hydroxy-2-nonenal, a specific lipid peroxidation product, is elevated in lungs of patients with chronic obstructive pulmonary disease. Am. J. Respir. Crit Care Med..

[25] Liu J., Huang J., Liu H., Chen C., Xu J., Zhong L. (2021). Elevated serum 4HNE plus decreased serum thioredoxin: Unique feature and implications for acute exacerbation of chronic obstructive pulmonary disease. PLoS ONE.

[26] Birch J., Barnes P.J., Passos J.F. (2018). Mitochondria, telomeres and cell senescence: Implications for lung ageing and disease. Pharmacol. Ther..

[27] Cloonan S.M., Kim K., Esteves P., Trian T., Barnes P.J. (2020). Mitochondrial dysfunction in lung ageing and disease. Eur. Respir. Rev..

[28] Van Der Toorn M., Rezayat D., Kauffman H.F., Bakker S.J., Gans R.O., Koëter G.H., Choi A.M.K., Van Oosterhout A.J.M., Slebos D.J. (2009). Lipid-soluble components in cigarette smoke induce mitochondrial production of reactive oxygen species in lung epithelial cells. Am. J. Physiol. Lung Cell Mol. Physiol..

[29] Keatings V.M., Barnes P.J. (1997). Granulocyte activation markers in induced sputum: Comparison between chronic obstructive pulmonary disease, asthma and normal subjects. Am. J. Respir Crit. Care Med..

[30] Osoata G.O., Hanazawa T., Brindicci C., Ito M., Barnes P.J., Kharitonov S., Ito K. (2009). Peroxynitrite elevation in exhaled breath condensate of COPD and its inhibition by fudosteine. Chest.

[31] O’Donnell C., Newbold P., White P., Thong B., Stone H., Stockley R.A. (2010). 3-Chlorotyrosine in sputum of COPD patients: Relationship with airway inflammation. COPD.

[32] Negre-Salvayre A., Coatrieux C., Ingueneau C., Salvayre R. (2008). Advanced lipid peroxidation end products in oxidative damage to proteins. Potential role in diseases and therapeutic prospects for the inhibitors. Br. J. Pharmacol.

[33] Kirkham P.A., Caramori G., Casolari P., Papi A., Edwards M., Shamji B., Triantaphyllopoulos K., Hussain F., Pinart M., Khan Y. (2011). Oxidative stress-induced antibodies to carbonyl-modified protein correlate with severity of COPD. Am. J. Respir. Crit. Care Med..

[34] Paredi P., Kharitonov S.A., Leak D., Ward S., Cramer D., Barnes P.J. (2000). Exhaled ethane, a marker of lipid peroxidation, is elevated in chronic obstructive pulmonary disease. Am. J. Respir. Crit. Care Med..

[35] Bartoli M.L., Novelli F., Costa F., Malagrino L., Melosini L., Bacci E., Cianchetti S., Dente F.L., Di Franco A., Vagaggini B. (2011). Malondialdehyde in exhaled breath condensate as a marker of oxidative stress in different pulmonary diseases. Mediat. Inflamm..

[36] Corradi M., Pignatti P., Manini P., Andreoli R., Goldoni M., Poppa M., Moscato G., Balbi B., Mutti A. (2004). Comparison between exhaled and sputum oxidative stress biomarkers in chronic airway inflammation. Eur. Respir. J..

[37] Dekhuijzen P.N.R., Aben K.H.H., Dekker I., Aarts L.P.H.J., Wielders P.L.M., van Herwarden C.L.A., Bast A. (1996). Increased exhalation of hydrogen peroxide in patients with stable and unstable chronic obstructive pulmonary disease. Am. J. Respir. Crit. Care Med..

[38] Montuschi P., Collins J.V., Ciabattoni G., Lazzeri N., Corradi M., Kharitonov S.A., Barnes P.J. (2000). Exhaled 8-isoprostane as an in vivo biomarker of lung oxidative stress in patients with COPD and healthy smokers. Am. J. Respir Crit. Care Med..

[39] Biernacki W.A., Kharitonov S.A., Barnes P.J. (2003). Increased leukotriene B4 and 8-isoprostane in exhaled breath condensate of patients with exacerbations of COPD. Thorax.

[40] Paredi P., Kharitonov S.A., Barnes P.J. (2002). Analysis of expired air for oxidation products. Am. J. Respir. Crit. Care Med..

[41] Ichinose M., Sugiura H., Yamagata S., Koarai A., Shirato K. (2000). Increase in reactive nitrogen species production in chronic obstructive pulmonary disease airways. Am. J. Resp. Crit Care Med..

[42] Ricciardolo F.L., Caramori G., Ito K., Capelli A., Brun P., Abatangelo G., Papi A., Chung K.F., Adcock I., Barnes P.J. (2005). Nitrosative stress in the bronchial mucosa of severe chronic obstructive pulmonary disease. J. Allergy Clin. Immunol..

[43] Barreiro E., Peinado V.I., Galdiz J.B., Ferrer E., Marin-Corral J., Sanchez F., Gea J., Barbera J.A. (2010). Cigarette smoke-induced oxidative stress: A role in chronic obstructive pulmonary disease skeletal muscle dysfunction. Am. J. Respir. Crit. Care Med..

[44] Drost E.M., Skwarski K.M., Sauleda J., Soler N., Roca J., Agusti A., Macnee W. (2005). Oxidative stress and airway inflammation in severe exacerbations of COPD. Thorax.

[45] Sorheim I.C., DeMeo D.L., Washko G., Litonjua A., Sparrow D., Bowler R., Bakke P., Pillai S.G., Coxson H.O., Lomas D.A. (2010). Polymorphisms in the superoxide dismutase-3 gene are associated with emphysema in COPD. COPD J. Chronic. Obstr. Pulm. Dis..

[46] Yao H., Arunachalam G., Hwang J.W., Chung S., Sundar I.K., Kinnula V.L., Crapo J.D., Rahman I. (2010). Extracellular superoxide dismutase protects against pulmonary emphysema by attenuating oxidative fragmentation of ECM. Proc. Natl. Acad. Sci. USA.

[47] Xu J., Li T., Wu H., Xu T. (2012). Role of thioredoxin in lung disease. Pulm. Pharmacol. Ther..

[48] Liu Q., Gao Y., Ci X. (2019). Role of Nrf2 and its activators in respiratory diseases. Oxidative Med. Cell. Longev..

[49] Mercado N., Thimmulappa R., Thomas C.M., Fenwick P.S., Chana K.K., Donnelly L.E., Biswal S., Ito K., Barnes P.J. (2011). Decreased histone deacetylase 2 impairs Nrf2 activation by oxidative stress. Biochem. Biophys. Res. Commun..

[50] Hwang J.W., Rajendrasozhan S., Yao H., Chung S., Sundar I.K., Huyck H.L., Pryhuber G.S., Kinnula V.L., Rahman I. (2011). FOXO3 deficiency leads to increased susceptibility to cigarette smoke-induced inflammation, airspace enlargement, and chronic obstructive pulmonary disease. J. Immunol..

[51] Mizumura K., Gon Y. (2021). Iron-regulated reactive oxygen species production and programmed cell death in chronic obstructive pulmonary disease. Antioxidants.

[52] Perez E., Baker J.R., Di Giandomenico S., Kermani P., Parker J., Kim K., Yang J., Barnes P.J., Vaulont S., Scandura J.M. (2020). Hepcidin Is essential for alveolar macrophage function and is disrupted by smoke in a murine Chronic Obstructive Pulmonary Disease model. J. Immunol..

[53] Rahman I., Adcock I.M. (2006). Oxidative stress and redox regulation of lung inflammation in COPD. Eur. Respir. J..

[54] McGuinness A.J., Sapey E. (2017). Oxidative stress in COPD: Sources, markers, and potential mechanisms. J. Clin. Med..

[55] Barnes P.J. (2009). The cytokine network in COPD. Am. J. Respir. Cell. Mol. Biol..

[56] Caramori G., Romagnoli M., Casolari P., Bellettato C., Casoni G., Boschetto P., Fan C.K., Barnes P.J., Adcock I.M., Ciaccia A. (2003). Nuclear localisation of p65 in sputum macrophages but not in sputum neutrophils during COPD exacerbations. Thorax.

[57] Gorowiec M.R., Borthwick L.A., Parker S.M., Kirby J.A., Saretzki G.C., Fisher A.J. (2012). Free radical generation induces epithelial-to-mesenchymal transition in lung epithelium via a TGF-beta1-dependent mechanism. Free Radic. Biol. Med..

[58] Aschner Y., Downey G.P. (2016). Transforming growth factor-β: Master regulator of the respiratory system in health and disease. Am. J. Respir. Cell Mol. Biol..

[59] Michaeloudes C., Sukkar M.B., Khorasani N.M., Bhavsar P.K., Chung K.F. (2011). TGF-beta regulates Nox4, MnSOD and catalase expression, and IL-6 release in airway smooth muscle cells. Am. J. Physiol. Lung Cell Mol. Physiol..

[60] Taggart C., Cervantes-Laurean D., Kim G., McElvaney N.G., Wehr N., Moss J., Levine R.L. (2000). Oxidation of either methionine 351 or methionine 358 in alpha 1-antitrypsin causes loss of anti-neutrophil elastase activity. J. Biol. Chem..

[61] Barnes P.J. (2013). Corticosteroid resistance in patients with asthma and chronic obstructive pulmonary disease. J. Allergy Clin. Immunol..

[62] To Y., Ito K., Kizawa Y., Failla M., Ito M., Kusama T., Elliot M., Hogg J.C., Adcock I.M., Barnes P.J. (2010). Targeting phosphoinositide-3-kinase-d with theophylline reverses corticosteroid insensitivity in COPD. Am. J. Resp. Crit. Care Med..

[63] Osoata G., Yamamura S., Ito M., Vuppusetty C., Adcock I.M., Barnes P.J., Ito K. (2009). Nitration of distinct tyrosine residues causes inactivation of histone deacetylase 2. Biochem. Biophy. Res. Commun..

[64] Ito K., Yamamura S., Essilfie-Quaye S., Cosio B., Ito M., Barnes P.J., Adcock I.M. (2006). Histone deacetylase 2-mediated deacetylation of the glucocorticoid receptor enables NF-kB suppression. J. Exp. Med..

[65] Mitani A., Ito K., Vuppusetty C., Barnes P.J., Mercado N. (2016). Restoration of corticosteroid sensitivity in chronic obstructive pulmonary disease by inhibition of mammalian target of rapamycin. Am. J. Respir. Crit. Care Med..

[66] Houssaini A., Breau M., Kebe K., Abid S., Marcos E., Lipskaia L., Rideau D., Parpaleix A., Huang J., Amsellem V. (2018). mTOR pathway activation drives lung cell senescence and emphysema. JCI Insight.

[67] Baker J., Vuppusetty C., Colley T., Papaioannou A., Fenwick P., Donnelly L., Ito K., Barnes P.J. (2016). Oxidative stress dependent microRNA-34a activation via PI3Kα reduces the expression of sirtuin-1 and sirtuin-6 in epithelial cells. Sci. Rep..

[68] Nakamaru Y., Vuppusetty C., Wada H., Milne J.C., Ito M., Rossios C., Elliot M., Hogg J., Kharitonov S., Goto H. (2009). A protein deacetylase SIRT1 is a negative regulator of metalloproteinase-9. FASEB J..

[69] Baker J., Vuppusetty C., Colley T., Hassibi S., Fenwick P.S., Donnelly Le Ito K., Barnes P.J. (2019). MicroRNA-570 is a novel regulator of cellular senescence and inflammaging. FASEB J..

[70] Martins W.K., Silva M., Pandey K., Maejima I., Ramalho E., Olivon V.C., Diniz S.N., Grasso D. (2021). Autophagy-targeted therapy to modulate age-related diseases: Success, pitfalls, and new directions. Curr. Res. Pharmacol. Drug. Discov..

[71] Racanelli A.C., Choi A.M.K., Choi M.E. (2020). Autophagy in chronic lung disease. Prog. Mol. Biol. Transl. Sci..

[72] Feghali-Bostwick C.A., Gadgil A.S., Otterbein L.E., Pilewski J.M., Stoner M.W., Csizmadia E., Zhang Y., Sciurba F.C., Duncan S.R. (2008). Autoantibodies in patients with chronic obstructive pulmonary disease. Am. J. Respir. Crit. Care Med..

[73] Byrne R., Todd I., Tighe P.J., Fairclough L.C. (2019). Autoantibodies in chronic obstructive pulmonary disease: A systematic review. Immunol. Lett..

[74] Caramori G., Adcock I.M., Casolari P., Ito K., Jazrawi E., Tsaprouni L., Villetti G., Civelli M., Carnini C., Chung K.F. (2011). Unbalanced oxidant-induced DNA damage and repair in COPD: A link towards lung cancer. Thorax.

[75] Adcock I.M., Caramori G., Barnes P.J. (2011). Chronic obstructive pulmonary disease and lung cancer: New molecular insights. Respiration.

[76] Fahy J.V., Dickey B.F. (2010). Airway mucus function and dysfunction. N. Eng. J. Med..

[77] Yuan S., Hollinger M., Lachowicz-Scroggins M.E., Kerr S.C., Dunican E.M., Daniel B.M., Ghosh S., Erzurum S.C., Willard B., Hazen S.L. (2015). Oxidation increases mucin polymer cross-links to stiffen airway mucus gels. Sci. Trans. Med..

[78] Dunican E.M., Elicker B.M., Henry T., Gierada D.S., Schiebler M.L., Anderson W., Barjaktarevic I., Barr R.G., Bleecker E.R., Boucher R.C. (2021). Mucus plugs and emphysema in the pathophysiology of airflow obstruction and hypoxemia in smokers. Am. J. Respir. Crit. Care Med..

[79] Thomson N.C. (2018). Targeting oxidant-dependent mechanisms for the treatment of respiratory diseases and their comorbidities. Curr. Opin. Pharmacol..

[80] Barnes P.J. (2020). Oxidative stress-based therapeutics in COPD. Redo. Biol..

[81] Kiyokawa H., Hoshino Y., Sakaguchi K., Muro S., Yodoi J. (2021). Redox regulation in aging lungs and therapeutic implications of antioxidants in COPD. Antioxidants.

[82] Tsiligianni I.G., Van der M.T. (2010). A systematic review of the role of vitamin insufficiencies and supplementation in COPD. Respir. Res..

[83] Biswas S., Hwang J.W., Kirkham P.A., Rahman I. (2013). Pharmacological and dietary antioxidant therapies for chronic obstructive pulmonary disease. Curr. Med. Chem..

[84] Fischer A., Johansson I., Blomberg A., Sundstrom B. (2019). Adherence to a mediterranean-like diet as aprotective factor against COPD: A nested case-control study. COPD.

[85] Culpitt S.V., Rogers D.F., Fenwick P.S., Shah P., De Matos C., Russell R.E., Barnes P.J. (2003). Donnelly LE. Inhibition by red wine extract, resveratrol, of cytokine release by alveolar macrophages in COPD. Thorax.

[86] Birrell M.A., McCluskie K., Wong S., Donnelly L.E., Barnes P.J., Belvisi M.G. (2005). Resveratrol, an extract of red wine, inhibits lipopolysaccharide induced airway neutrophilia and inflammatory mediators through an NF-kB-independent mechanism. FASEB J..

[87] Navarro S., Reddy R., Lee J., Warburton D., Driscoll B. (2017). Inhaled resveratrol treatments slow ageing-related degenerative changes in mouse lung. Thorax.

[88] Bartholome A., Kampkotter A., Tanner S., Sies H., Klotz L.O. (2010). Epigallocatechin gallate-induced modulation of FoxO signaling in mammalian cells and *C. elegans*: FoxO stimulation is masked via PI3K/Akt activation by hydrogen peroxide formed in cell culture. Arch. Biochem. Biophys..

[89] Tian X., Xue Y., Xie G., Zhou Y., Xiao H., Ding F., Zhang M. (2021). Epicatechin ameliorates cigarette smoke-induced lung inflammation via inhibiting ROS/NLRP3 inflammasome pathway in rats with COPD. Toxicol. Appl. Pharmacol..

[90] Biswas S.K., Rahman I. (2009). Environmental toxicity, redox signaling and lung inflammation: The role of glutathione. Mol. Asp. Med..

[91] Grandjean E.M., Berthet P., Ruffmann R., Leuenberger P. (2000). Efficacy of oral long-term N-acetylcysteine in chronic bronchopulmonary disease: A meta-analysis of published double-blind, placebo-controlled clinical trials. Clin. Ther..

[92] Decramer M., Rutten-van Molken M., Dekhuijzen P.N., Troosters T., van Herwaarden C., Pellegrino R., Van Schayck C.P., Olivieri D., Del Donno M., De Backer W. (2005). Effects of N-acetylcysteine on outcomes in chronic obstructive pulmonary disease (Bronchitis Randomized on NAC Cost-Utility Study, BRONCUS): A randomised placebo-controlled trial. Lancet.

[93] Zheng J.P., Wen F.Q., Bai C.X., Wan H.Y., Kang J., Chen P., Yao W.Z., Ma L.J., Li X., Raiteri L. (2014). Twice daily N-acetylcysteine 600 mg for exacerbations of chronic obstructive pulmonary disease (PANTHEON): A randomised, double-blind placebo-controlled trial. Lancet Resp. Med..

[94] Papi A., Zheng J., Criner G.J., Fabbri L.M., Calverley P.M.A. (2019). Impact of smoking status and concomitant medications on the effect of high-dose N-acetylcysteine on chronic obstructive pulmonary disease exacerbations: A post-hoc analysis of the PANTHEON study. Respir. Med..

[95] Zheng J.P., Kang J., Huang S.G., Chen P., Yao W.Z., Yang L., Bai C.X., Wang C.Z., Wang C., Chen B.Y. (2008). Effect of carbocisteine on acute exacerbation of chronic obstructive pulmonary disease (PEACE Study): A randomised placebo-controlled study. Lancet.

[96] Zeng Z., Yang D., Huang X., Xiao Z. (2017). Effect of carbocisteine on patients with COPD: A systematic review and meta-analysis. Int. J. COPD.

[97] Calverley P.M., Page C., Dal Negro R.W., Fontana G., Cazzola M., Cicero A.F., Pozzi E., Wedzicha J.A. (2019). Effect of erdosteine on COPD exacerbations in COPD patients with moderate airflow limitation. Int. J. COPD.

[98] Dal Negro R.W., Wedzicha J.A., Iversen M., Fontana G., Page C., Cicero A.F., Pozzi E., Calverley P.M.A. (2017). Effect of erdosteine on the rate and duration of COPD exacerbations: The RESTORE study. Eur. Respir. J..

[99] Poole P., Sathananthan K., Fortescue R. (2019). Mucolytic agents versus placebo for chronic bronchitis or chronic obstructive pulmonary disease. Cochrane Database Syst. Rev..

[100] Rogliani P., Matera M.G., Page C., Puxeddu E., Cazzola M., Calzetta L. (2019). Efficacy and safety profile of mucolytic/antioxidant agents in chronic obstructive pulmonary disease: A comparative analysis across erdosteine, carbocysteine, and N-acetylcysteine. Respir. Res..

[101] Day B.J. (2004). Catalytic antioxidants: A radical approach to new therapeutics. Drug Disc. Today.

[102] Foronjy R.F., Mirochnitchenko O., Propokenko O., Lemaitre V., Jia Y., Inouye M., Okada Y., D’Armiento J.M. (2006). Superoxide dismutase expression attenuates cigarette smoke- or elastase-generated emphysema in mice. Am. J. Respir. Crit. Care Med..

[103] Smith K.R., Uyeminami D.L., Kodavanti U.P., Crapo J.D., Chang L.Y., Pinkerton K.E. (2002). Inhibition of tobacco smoke-induced lung inflammation by a catalytic antioxidant. Free Rad. Biol. Med..

[104] Geraghty P., Hardigan A.A., Wallace A.M., Mirochnitchenko O., Thankachen J., Arellanos L., Thompson V., D’Armiento J.M., Foronjy R.F. (2013). The glutathione peroxidase 1-protein tyrosine phosphatase 1B-protein phosphatase 2A axis. A key determinant of airway inflammation and alveolar destruction. Am. J. Respir. Cell Mol. Biol..

[105] Brassington K., Chan S.M.H., Seow H.J., Dobric A., Bozinovski S., Selemidis S., Vlahos R. (2021). Ebselen reduces cigarette smoke-induced endothelial dysfunction in mice. Br. J. Pharmacol..

[106] Sies H., Berndt C., Jones D.P. (2017). Oxidative Stress. Annu. Rev. Biochem..

[107] Elbatreek M.H., Mucke H., Schmidt H. (2021). NOX inhibitors: From bench to naxibs to bedside. Handbk. Exp. Pharmacol..

[108] Oostwoud L.C., Gunasinghe P., Seow H.J., Ye J.M., Selemidis S., Bozinovski S., Vlahos R. (2016). Apocynin and ebselen reduce influenza A virus-induced lung inflammation in cigarette smoke-exposed mice. Sci. Rep..

[109] Stefanska J., Sarniak A., Wlodarczyk A., Sokolowska M., Doniec Z., Bialasiewicz P., Nowak D., Pawliczak R. (2012). Hydrogen peroxide and nitrite reduction in exhaled breath condensate of COPD patients. Pulm. Pharmacol. Ther..

[110] Zhu A., Ge D., Zhang J., Teng Y., Yuan C., Huang M., Adcock I.M., Barnes P.J., Yao X. (2014). Sputum myeloperoxidase in chronic obstructive pulmonary disease. Eur. J. Med. Res..

[111] Churg A., Marshall C.V., Sin D.D., Bolton S., Zhou S., Thain K., Cadogan E.B., Maltby J., Soars M.G., Mallinder P.R. (2012). Late intervention with a myeloperoxidase inhibitor stops progression of experimental COPD. Am. J. Respir. Crit. Care Med..

[112] Brindicci C., Kharitonov S.A., Ito M., Elliott M.W., Hogg J.C., Barnes P.J., Ito K. (2010). Nitric oxide synthase isoenzyme expression and activity in peripheral lungs of COPD patients. Am. J. Respir. Crit. Care Med..

[113] Seimetz M., Parajuli N., Pichl A., Veit F., Kwapiszewska G., Weisel F.C., Milger K., Egemnazarov B., Turowska A., Fuchs B. (2011). Inducible NOS inhibition reverses tobacco-smoke-induced emphysema and pulmonary hypertension in mice. Cell.

[114] Brindicci C., Ito K., Torre O., Barnes P.J., Kharitonov S.A. (2009). Effects of aminoguanidine, an inhibitor of inducible nitric oxide synthase, on nitric oxide production and its metabolites in healthy controls, healthy smokers and COPD patients. Chest.

[115] Cloonan S.M., Choi A.M. (2016). Mitochondria in lung disease. J. Clin. Invest..

[116] Wiegman C.H., Michaeloudes C., Haji G., Narang P., Clarke C.J., Russell K.E., Bao W., Pavlidis S., Barnes P.J., Kanerva J. (2015). Oxidative stress-induced mitochondrial dysfunction drives inflammation and airway smooth muscle remodeling in patients with chronic obstructive pulmonary disease. J. Allergy Clin. Immunol..

[117] Belchamber K.B.R., Singh R., Batista C.M., Whyte M.K., Dockrell D.H., Kilty I., Robinson M.J., Wedzicha J.A., Barnes P.J., Donnelly L.E. (2019). Defective bacterial phagocytosis is associated with dysfunctional mitochondria in COPD macrophages. Eur. Respir. J..

[118] Zinovkin R.A., Zamyatnin A.A. (2019). Mitochondria-targeted drugs. Curr. Mol. Pharmacol..

[119] Hara H., Araya J., Ito S., Kobayashi K., Takasaka N., Yoshii Y., Wakui H., Kojima J., Shimizu K., Numata T. (2013). Mitochondrial fragmentation in cigarette smoke-induced bronchial epithelial cell senescence. Am. J. Physiol. Lung Cell Mol. Physiol..

[120] Ryter S.W. (2022). Heme Oxygenase-1: An anti-inflammatory effector in cardiovascular, lung, and related metabolic disorders. Antioxidants.

[121] Even B., Fayad-Kobeissi S., Gagliolo J.M., Motterlini R., Boczkowski J., Foresti R., Dagouassat M. (2018). Heme oxygenase-1 induction attenuates senescence in chronic obstructive pulmonary disease lung fibroblasts by protecting against mitochondria dysfunction. Aging Cell.

[122] Lee J., Jang J., Park S.M., Yang S.R. (2021). An update on the role of Nrf2 in respiratory disease, Molecular mechanisms and therapeutic approaches. Int. J. Mol. Sci..

[123] Sussan T.E., Rangasamy T., Blake D.J., Malhotra D., El-Haddad H., Bedja D., Yates M.S., Kombairaju P., Yamamoto M., Liby K.T. (2009). Targeting Nrf2 with the triterpenoid CDDO-imidazolide attenuates cigarette smoke-induced emphysema and cardiac dysfunction in mice. Proc. Natl. Acad. Sci. USA.

[124] Rossing P. (2013). Diabetic nephropathy: Could problems with bardoxolone methyl have been predicted?. Nat. Rev. Nephrol..

[125] Gold R., Kappos L., Arnold D.L., Bar-Or A., Giovannoni G., Selmaj K., Tornatore C., Sweetser M.T., Yang M., Sheikh S.I. (2012). Placebo-controlled phase 3 study of oral BG-12 for relapsing multiple sclerosis. N. Engl. J. Med..

[126] Muralidharan P., Hayes D., Black S.M., Mansour H.M. (2016). Microparticulate/Nanoparticulate Powders of a Novel Nrf2 Activator and an aerosol performance enhancer for pulmonary delivery targeting the lung Nrf2/Keap-1 Pathway. Mol. Syst. Des. Eng..

[127] Jiang Z.Y., Lu M.C., Xu L.L., Yang T.T., Xi M.Y., Xu X.L., Guo X.K., Zhang X.J., You Q.D., Sun H.P. (2014). Discovery of potent Keap1-Nrf2 protein-protein interaction inhibitor based on molecular binding determinants analysis. J. Med. Chem..

[128] Mafra D., Alvarenga L., Cardozo L.F., Stockler-Pinto M.B., Nakao L.S., Stenvinkel P., Shiels P.G. (2022). Inhibiting BTB domain and CNC homolog 1 (Bach1) as an alternative to increase Nrf2 activation in chronic diseases. Biochim. Biophys. Acta.

